# The Outcomes of Robotic Rehabilitation Assisted Devices Following Spinal Cord Injury and the Prevention of Secondary Associated Complications

**DOI:** 10.3390/medicina58101447

**Published:** 2022-10-13

**Authors:** Carmen Delia Nistor-Cseppento, Anamaria Gherle, Nicoleta Negrut, Simona Gabriela Bungau, Anca Maria Sabau, Andrei-Flavius Radu, Alexa Florina Bungau, Delia Mirela Tit, Bogdan Uivaraseanu, Timea Claudia Ghitea, Diana Uivarosan

**Affiliations:** 1 Department of Psycho Neuroscience and Recovery, Faculty of Medicine and Pharmacy, University of Oradea, 410073 Oradea, Romania; 2 President Medcenter Baile Felix, 417500 Baile Felix, Romania; 3 Doctoral School of Biological and Biomedical Sciences, University of Oradea, 410087 Oradea, Romania; 4 Department of Pharmacy, Faculty of Medicine and Pharmacy, University of Oradea, 410028 Oradea, Romania; 5 Department of Physical Education, Sport and Physical Therapy, Faculty of Geography, Tourism and Sport, University of Oradea, 410087 Oradea, Romania; 6 Department of Preclinical Disciplines, Faculty of Medicine and Pharmacy, University of Oradea, 410073 Oradea, Romania

**Keywords:** spinal cord injuries, associated complications, robotic devices, neuro-motor rehabilitation, recovery

## Abstract

Spinal cord injuries (SCIs) have major consequences on the patient’s health and life. Voluntary muscle paralysis caused by spinal cord damage affects the patient’s independence. Following SCI, an irreversible motor and sensory deficit occurs (spasticity, muscle paralysis, atrophy, pain, gait disorders, pain). This pathology has implications on the whole organism: on the osteoarticular, muscular, cardiovascular, respiratory, gastrointestinal, genito-urinary, skin, metabolic disorders, and neuro-psychic systems. The rehabilitation process for a subject having SCIs can be considered complex, since the pathophysiological mechanism and biochemical modifications occurring at the level of spinal cord are not yet fully elucidated. This review aims at evaluating the impact of robotic-assisted rehabilitation in subjects who have suffered SCI, both in terms of regaining mobility as a major dysfunction in patients with SCI, but also in terms of improving overall fitness and cardiovascular function, respiratory function, as well as the gastrointestinal system, bone density and finally the psychosocial issues, based on multiple clinical trials, and pilot studies. The researched literature in the topic revealed that in order to increase the chances of neuro-motor recovery and to obtain satisfactory results, the combination of robotic therapy, a complex recovery treatment and specific medication is one of the best decisions. Furthermore, the use of these exoskeletons facilitates better/greater autonomy for patients, as well as optimal social integration.

## 1. Introduction

Spinal cord injuries (SCIs) have a significant negative effect on patients’ quality of life. Voluntary muscle paralysis caused by spinal cord damage affects the patient’s independence [1]. Following SCI, an irreversible motor and sensory deficit occurs (spasticity, muscle paralysis, atrophy, pain, gait disorders, pain) [2]. This pathology has implications on the whole organism: on the osteoarticular, muscular, cardiovascular, respiratory, gastrointestinal, genito-urinary, skin, metabolic disorders and neuro-psychic systems [3]. 

The challenges that a patient with damage to the spinal cord faces are very complex and need a multidisciplinary approach, therefore the bodily changes, functional dependence, impaired mobility, and incontinence all constitute overwhelming losses that most of the patients come to term [4]. 

Restoring and regaining the patient’s abilities is the main goal of neurohabilitation management, thus contributing to increasing functional independence and quality of life. [1,5]. Due to the fact that the physio pathological processes and biochemical pathways in the spinal cord are still poorly understood, the rehabilitation intervention for a patient suffering SCIs is comprehensive and challenging [6]. The already published obtained results underline the main benefits of these robotic therapies in question, in SCI subjects.

Within this quite narrow field, the present review aims at evaluating the impact of robotic technology rehabilitation in patients with SCIs, both with respect to regaining mobility as a major dysfunction in patients with SCI, but also in terms of improving overall fitness and cardiovascular function, respiratory function, as well as the gastrointestinal system, bone density and finally the psychosocial issues, based on multiple clinical trials, and pilot studies. 

## 2. Methodology

**Figure 1:**
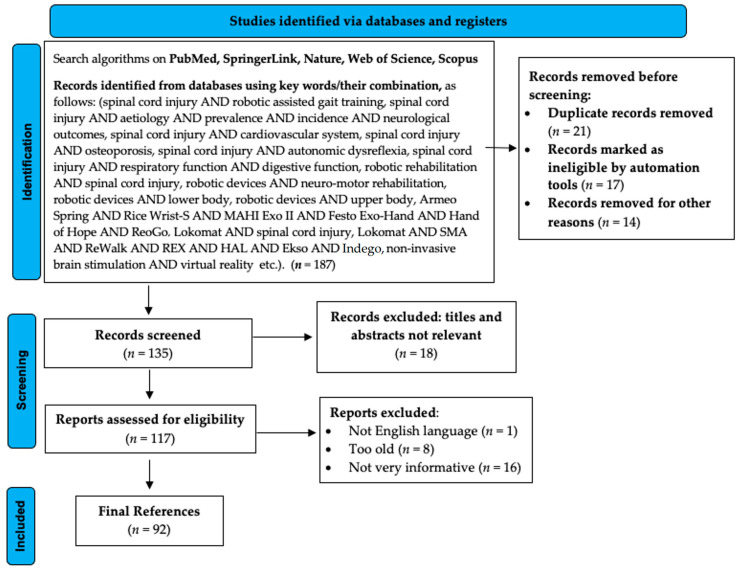
Literature selection depicted in a PRISMA 2020 flow diagram.

This review paper identified and centralized scientific publications that assessed robotic rehabilitation assisted devices following SCI and the implications on other body systems between 1996 and 2022. The conceptual design and methodological approaches involve a complex search of the scientific literature dictated by a predetermined algorithm. In this regard, extensive scientific databases that span a wide range of medical issues were used to perform medical literature research (e.g., PubMed, SpringerLink, Nature, Web of Science, Scopus). In addition, the application of the search algorithm in the PubMed database involved also the use of a thesaurus of controlled vocabulary (e.g., medical subject titles). In the final, 92 bibliographic references were selected and evaluated to validate the scientific data presented in this paper. The steps of literature selection have been presented in Figure 1, which was realized according to Page et al.’s indications [7]. 

## 3. Spinal Cord Injury: Etiology, Neurological Outcomes, and Treatment

### 3.1. Etiology

Infections, tumors, or trauma cause SCIs. Regardless of the etiology, it determines the loss of the lesion’s distal function and/or and/or sensory), being classified as complete or incomplete [6].

Traffic crashes, bullet wounds, blunt force trauma, falls, and sports incidents constitute the most frequent leading causes of SCIs (90%) [6]. 

Over time, there have been significant variations in terms of etiology; acts of violence that produced injuries to the spinal cord reached their peak around the 90s (21%), followed by a gradual decrease until 2000. Injuries to the spinal cord through sports trauma and road accidents remained approximately constant [8]. Due to the musculoskeletal modifications that occur with aging, the incidence of SCI by falls increased from 16.2% to 21.8% (in the year 2000) as life expectancy and average population age grew worldwide [6,8].

External mechanisms of SCI are flexion, compression, hyperextension, or flexion rotation [4].

Patients with SCIs typically experience significant neurological impairments and disabilities that are persistent.

### 3.2. Neurological Outcomes of SCI

Depending on the injury level and the damage to the spinal cord, the patients can be diagnosed with either tetraplegia or paraplegia. Tetraplegia or quadriplegia refers to the loss of motor and/or sensory function in both the upper and lower body due to a lesion of the cervical segments of the spinal cord [6]. Lower thoracic and lumbar injuries can cause paraplegia [1]. The lesion levels and the functional deficit are included in Table 1. 

### 3.3. Treatment

Early rehabilitation after neurological stabilization (6–12 weeks) is essential to prevent contractures and retractions, decrease muscle strength in the unaffected groups, and decrease bone mass loss. Every 2–3 h, the patient’s posture in bed is switched to avoid pressure sores. It is also necessary to maintain the function of the affected respiratory, cardiovascular, and digestive systems. It is carried out through postures (with bags, pillows, orthoses), passive mobilization of large joints, throughout the range of motion, 1–2 times a day, during the flaccid period and three times a day after the onset of spasticity (after the shock phase spinal) [1]. Along with passive mobilizations, muscle stretching is recommended to prevent tenodesis, especially at the level of the fingers. An essential role in recovering patients with SCI is occupied by breathing exercises to preserve respiratory function and toning the muscles of the upper limbs (especially in paraplegics to increase independence). Free active mobilizations with progressive resistance are recommended. Muscle electrostimulation is also helpful [9]. 

In the chronic phase, the main objective is the recovery of walking. This aspect is strictly related to the patient’s age, lesion level, general condition and comorbidities, motivation, and status of spasticity. For example, patients with lesion levels below T11-L2 can move short distances (domestic level), while those with damage below L2 can perform social walking.

In the last 15 years, exoskeletons have made their way into motor recovery, facilitating walking, and taking the place of passive orthoses. They can be used in SCI, stroke [10,11], or other neurological disorders, promoting functional walking (with average speeds of 0.26–0.42 m/s) [12]. 

The first three months following the traumatic event are when motor rehabilitation is most significant. Furthermore, after nine months, there is a plateau phase in recovery, which is followed by a gradual phase of recovery lasting up to a year and a half [6]. The essential predictor in functional recovery is the presence of complete or incomplete injury.

## 4. Robotic Devices Used in Neuro-Motor Rehabilitation

At the end of the 19th century, the first assisted walking devices or "exoskeletons" used for walking recovery were patented, but only after a century could they be used in practice [13].

Unfortunately, there is no way to reverse damage to the spinal cord. Therefore, the goal of treatment is to prevent further damage from occurring while also enhancing the patients’ life quality. The main objective is to restore motor function; in practice, in addition to conventional therapy, robotic systems are used, classified as fixed exercise robots for limbs or robotic orthoses, which the patient wears [14].

These robotic devices are based on repetitive movement exercise, promoting functional recovery through moving further than orthoses and wheelchairs to incorporate neuroplasticity as mobility assistance [15,16]. After evaluating the functional deficit, robotic therapy is prescribed. Table 2 includes the possibilities of ambulation depending on the lesion level.

### 4.1. Rehabilitation Devices for Upper Body

**Figure 2:**
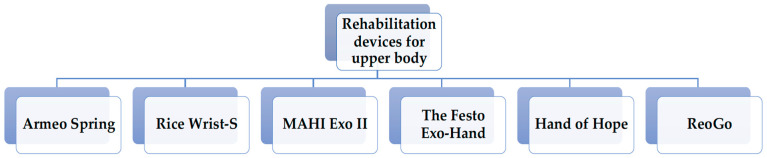
Exoskeleton used in upper limb rehabilitation. Armeo Spring (AS)—Limitations of Conventional Arm and Hand Therapy; Rice Wrist-5 (RW)—Rice-body formation and tenosynovitis of the wrist; MAHI Exo II (E II)—Robotic Exoskeleton for Upper Extremity Rehabilitation; The Festo Exo-Hand (TFEH)—TFEH from Festo is an exoskeleton that can be worn like a glove; Hand of Hope (HoH)—HoH therapy device is intended for use in patients that require hand and forearm rehabilitation; ReoGo—Robotic Rehabilitation treatment for stroke.

Robotic devices have been shown to be effective in addition to classical ones for the motor dysfunctions in the upper extremities following SCIs. Over time, a series of exoskeletons were built that were used to rehabilitate several diseases involving functional deficits in the upper limbs due to neurological causes. Some of the devices described in the literature are presented in Figure 2. Most devices are static, with varying degrees of freedom. They are used in specialized recovery centres. Research continues to design exoskeletons to be used at home to obtain maximum autonomy [17]. 

### 4.2. Rehabilitation Devices for Lower Body

**Figure 3:**
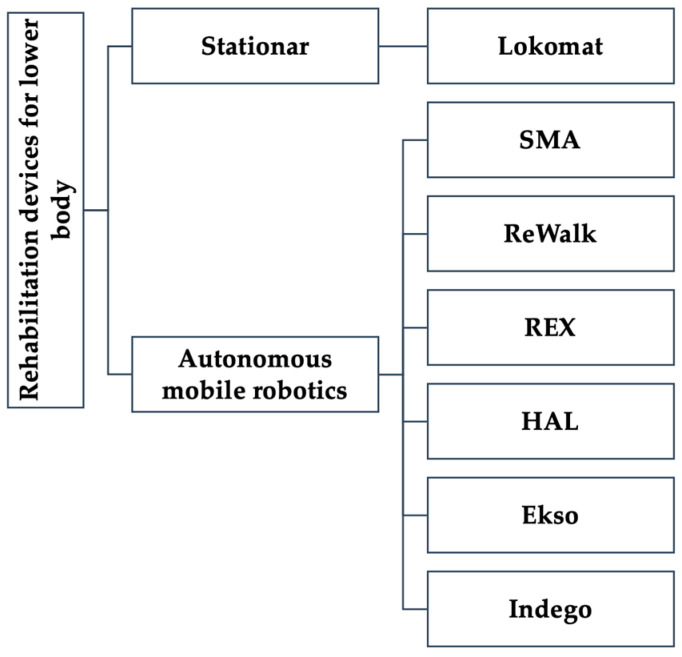
Robotic devices are used for lower limb rehabilitation. Lokomat—Robot-assisted therapy enables effective and intensive training; SMA (Shape Memory Alloy); ReWalk (Robotics, Yokneam, Israel)—ReWalk is a wearable robotic exoskeleton that provides powered hip and knee motion to enable individuals with spinal cord injury (SCI); REX (Rex Bionics PLC, London, UK)—mission is to develop technology in the field of autonomous mobile robotics, role in uprighting and walking training for patients with complete or incomplete C4-L5 injury; HAL (Hybrid Assistive Limb)—an exoskeleton for uprighting and training is walking; Ekso (Ekso Bionics, Richmond CA, USA)—an exoskeleton for training walking; Indego (Parker Hannifin, OH, USA)—The Indego is a powered hip-knee exoskeleton for gait training by motion.

In practice, at the moment, there is a wide variety of exoskeletons, stationary (Lokomat) or autonomous support systems for hip and knee, hip-knee-ankle, or a single joint. A systematic review (2021) identified 25 autonomous exoskeletons, of which only 6 had FDA approval (Ekso, HAL, Indego, REX, ReWalk and SMA) [18] (Figure 3) [3]. 

Exoskeletons increase mobility, improve the limb’s motor function, and restore the regular walking pattern [19].

## 5. Extensive Methods of Neuro-Motor Rehabilitation

**Figure 4:**
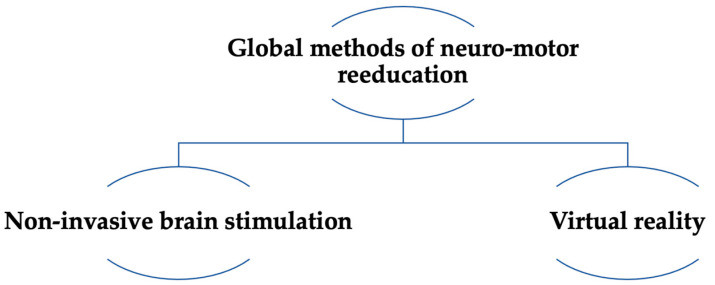
Global methods of neuro-motor rehabilitation.

Global methods of neuro-motor rehabilitation are currently in use alongside exoskeletons for upper and lower limbs, and gait re-education (Figure 4). 

### 5.1. Virtual Reality

A tool that can be utilized to train the upper and lower limbs is virtual reality (VR). To complete the task specified by the device, each patient should perform a variety of movements. As it gets results it appears as instant visual and audio feedback [20]. The benefits of this therapy, based on games and movements found in everyday life, are relearning and regaining lost skills and physical functions [21]. VR is therefore an additional therapy for patients with SCI who are recovering their mobility. It is considered that the motor relearning that is obtained through repeated exercises in the digital infrastructure is founded on neuroplasticity [22].

### 5.2. Non-Invasive Brain Stimulation

Focusing on the regulation of neuroplasticity, non-invasive brain stimulation techniques (NIBS) are applied for neurocognitive or neuropsychiatric rehabilitation. [23,24]. 

Both transcranial direct current stimulation (tDCS) and transcranial magnetic stimulation (TMS) are implemented in a clinical context. TMS consists of the application of a magnetic field that, by electromagnetism, causes an electrostatic potential in the brain. Electrical impulses are triggered by the generated electric field, which also modulates neuronal function. The location of the coil, the stimulation’s amplitude, regularity, and quantity of pulses all impact the effects of TMS differently based on the location of stimulation. tDCS is a form of transcranial electrical stimulation (TES) that involves applying a steady, weak current (1–2 mA) to the cortex via electrodes placed on the scalp and correlating to a particular cortical area. Moreover, this regulation, which is polarity-dependent, entails a transition from cathodal activation to hyperpolarization or from anodal stimulation to depolarization [25,26]. 

## 6. SCI-Associated Complications

Health issues like fever, pressure sores, respiratory disorders, deep vessel thrombosis, electrolyte imbalances, contractions, soreness, calcifying myositis, bladder infections, cardiac disorders, autonomic dysfunction, osteoporosis, bone fractures, and rashes are frequent complications in SCI patients [26]. 

### 6.1. Cardiovascular System

The primary cause of death in both the overall population and SCI patients is cardiovascular pathology. Patients with SCIs present a 60–70% prevalence of asymptomatic cardiovascular disease. Furthermore, patients with SCI show a 30–50% prevalence of exhibiting symptomatic cardiovascular events, compared to 5–10% in the overall population [27]. Patients with thoracic and cervical lesion levels show hemodynamic instability [28]. The cardiovascular complications associated with SCI are: orthostatic hypotension, thromboembolism, autonomic dysreflexia and precordial pain [29]. Verticalization of the patient with SCI can cause hypotension; therefore, testing is recommended during verticalization. The decrease in physical activity, through the appearance of motor deficits, is the first cause of the premature death of these patients. This risk factor (lack of physical activity) will have other consequences on the metabolism (lipid, carbohydrate and protein), especially during periods of growth [27]. Whether patients who use robotic devices can perform training at a sufficiently high intensity to obtain effects on general fitness arises [30]. 

After the SCI, the thoracolumbar spine and brain stem’s ability to communicate (the sympathetic flow’s origin is at the T1–L2 level), is interrupted; the parasympathetic nervous system’s implications persist [31]. Moreover, intrinsic imbalance between sympathetic and parasympathetic control causes bradycardia and bradyarrhythmia, resulting in arterial hypotension. Patients with lesions above T6 may present with autonomic dysreflexia which can be manifested by mild symptoms (headaches, sweating, piloerection and anxiety) or severe (rhythm disturbances, HTN with values above 300 mm Hg) [32]. After the approval of robotic devices, the benefits of robotic rehabilitation on cardiac health in individuals with SCIs were assessed. The study conducted by Touriel et al. (2011), on 14 patients, sought to ascertain the impact of resistance training combined with robotics training (supported body weight). It has been evaluated the left ventricular systolic-diastolic function, coronary flow reserve and endothelial function [33]. The findings indicated that following six weeks of therapy, the sensory-motor function improved in the left ventricle and the endothelial one was reduced inflammatory status. The study conducted on 13 patients with complete SCI (2018) showed an increase in O2 consumption, respiratory exchange ratio and heart rate both when the patient was upright and during walking, conditioned by the intensity of the effort [34].

### 6.2. Autonomic Dysreflexia

Injury to the spinal marrow impacts the propagation of the nerve impulses at the level of the descending bulbospinal tracts, which inhibits the sympathetic spinal reflexes, a mechanism involved in triggering autonomic dysreflexia. The supraspinal autonomic system is affected [35]. It is characteristic of the triad: sweating, arterial hypertension with bradycardia. It appears in the case of lesion levels above T6. High blood pressure values (>220 mm Hg) may favor intracerebral hemorrhage. Other complications that may occur are acute gastric or duodenal ulcers and motility disorders at the level of the colon and the level of the sphincter muscles of the urinary bladder [36]. The prevention of this complication costs the control of the triggering factors and monitoring of the voltage values.

### 6.3. Bone Mineral Density

Osteoporosis below the lesion level is a complication that frequently occurs shortly after the appearance of the SCI, increasing vulnerability to fractures (double compared to the general population) [37]. The cause is multifactorial: mechanical unloading, neuro-hormonal imbalance, and changes in bone vascularization [38,39]. The loss of bone mass is evident through osteo-densitometry measurements and the measurement of bone resorption markers (maximum levels obtained 10–16 weeks after the traumatic event) [40]. Bone mass continues to decrease, at a slower rate. The effect study on 39 patients (2008) supports a limitation of BMD loss after 30 months [41]. A study published in 1999 [42], (performed on eight pairs of monozygotic twins), supports the continuous trend of decreasing bone mass. Another study, carried out on 204 patients (1997) supports the stabilization of bone mass after 19 years [43].

In quadriplegics, BMD is lower in the lumbar spine and the upper limbs than in paraplegics, but the bone density is similar in the lower limbs. BMD is lower in cases with complete injury than in those with incomplete injury [44]. The decrease in bone mass in patients with SCI is not influenced by age and sex [42,45]. Two studies (1998, 2005) mentioned the possibility of the influence of spasticity on BMD, emphasizing the fact that spastic patients have a higher BMD than those who are flabby [44,46].

The review published in 2021, which followed the effects of various therapeutic interventions on osteoporosis given by SCI, did not identify improvements in the osteoporosis T-score (11 studies out of 16), regardless of whether the physical training was conventional or with exoskeletons on the treadmill or autonomous; 4 studies supported the acute phase mitigation of bone density reduction and the improvement of bone mass in the chronic phase by verticalization and walking on the treadmill supported by a robotic device (study conducted on patients who exclusively used the wheelchair) [40]. 

### 6.4. Respiratory Recovery

Along with cardiac damage, respiratory dysfunction is the leading cause of mortality and morbidity [8]. Particularly within the initial year following the accident, respiratory issues are typically the cause of mortality [47]. 

At the cervical level (C3–C4) originates the phrenic nerve that innervates the diaphragm (responsible for changing respiratory volumes at the ribcage level Hoh et al., 2013). The nerves that innervate the inspiratory and expiratory muscles will additionally be impacted if the upper cervical spinal cord is injured, potentially causing breathing impairment up to apnea and other respiratory complications [48]. Some functional plasticity is mentioned, but the functional deficit persists for a long time. The respiratory evaluation measures the tidal volume, FEV1, and the activity of the respiratory nerves through EMG. Published studies show the importance of early initiation of physical training to prevent lung function decline.

The effects of training with robotic devices on the respiratory system are evident in improving aerobic fitness. The 2014 study, conducted on ten patients who followed a robot-assisted walking training program consisting of 24 sessions (moderate effort > 3MET and low effort < 3MET), demonstrated a lower heart rate at rest and in the submaximal effort, resulting in an improvement in cardiorespiratory fitness. 

The comparative study (2019) carried out on 88 patients, who benefited from conventional and, respectively, conventional treatment associated with robotic therapy, demonstrated the superiority of the results in terms of the patient’s functional capacity in the case of combined therapy. The walking index and independence measurement score were higher in the case of the group that followed 16 sessions of robotic treatment (in 8 weeks) and conventional therapy five days/week [14].

### 6.5. Intestinal Function

Paraplegia, apart from motor dysfunction, is associated with urinary and anal sphincter dysfunction [1]. The systematic review published in 2022 that assessed the impact of robotics technology on systems in individuals with SCI, suggested the impact of this therapy on the neurogenic colon (referenced by 8 studies out of 41 validated). Autonomous exoskeletons such as Ekso, Indego, and ReWalk were used in the treatment. The results show an improvement in intestinal function [3]. 

The meta-analysis that included 111 patients with SCI indicates an improvement in intestinal function in 61% of the evaluated subjects [14]. 

### 6.6. Neurogenic Bladder

Life satisfaction is lowered by the absence of sphincter control. Damage to the spinal marrow above S1 causes reflex bladder dysfunction. The external sphincter and urinary detrusor musculature exhibit hypertonic and uncontrolled contractions. The micturition reflex disappears. The treatment consists of periodic polling and the administration of anticholinergic medication. To ensure patient safety, individuals should receive bladder management data as promptly as possible after SCI. Credé and Valsalva manoeuvres can be used to evacuate the bladder [36].

### 6.7. Erectile Dysfunction

The effect of SCIs on erectile activity is correlated with the injury’s level and severity. The restoration of sexual potential during the patient’s recovery period with SCI is mentioned [49]. Studies were not found showing the direct benefits of robot-assisted therapy on bladder and erectile dysfunction.

### 6.8. Psychosocial Adjustment

Pain and depression are complications of SCI [50]. The suicide rate increases in patients under 55 [1]. Standardized tests to evaluate the psychosocial impact in patients with SCI following robot-assisted training are lacking. Furthermore, the application of the Psychosocial Impact of the Assistive Device Scale (PIADS) on a sample of 10 patients demonstrated the benefits of training with Lokomat in patients with an impaired walking motor on the psychological aspect in addition to the motor benefits [51]. The absence of daily activity, and non-involvement in social life lead to depression, alcohol addiction and suicide [1].

### 6.9. Neuropathic Pain

Peripheral neuropathy affects 80% of patients, and 33% of the individuals are unresponsive to therapy [52]. Pozeg et al. (2017) evaluated the effects of VR on psychological aspects and neuropathic pain. The study was performed on 20 patients with SCI; the control group consisted of 20 healthy patients. The study’s conclusions showed an improvement in motivation, neuropathic pain, balance, and mobility under the action of multisensory stimulation [53]. A review published in 2021 (9 studies), which included 207 patients, supports significant results of VR on neuropathic pain [54].

## 7. Effects of Robotic Devices on SCI-Associated Complications

Exoskeletons have a role in the effect of propulsion and discharge of body weight. The verticalization and loading of the lower limbs are essential for the sensory stimulation of the proprioceptors that will activate the medullary conduction pathways, even in patients with complete SCI (demonstrated by EMG) [55].

### 7.1. Effects on Gait

Locomotor Training aims to improve walking. It follows the principles of motor learning. It promotes sensory stimulation through verticalization, gradual loading of the lower limbs and hip extension. Reduces compensatory movements, maximizing recovery. In association, the balance of the upper limbs is promoted. Through all these mechanisms, the learning of physiological walking is favored. Gait training devices that are associated with VR increase patient motivation and participation. It has been proven that the effects on walking and balance are superior [55].

The longitudinal study published in 2015, in which nine patients participated (a control group of 14 patients), followed the effects of robot-assisted walking training. To explain the therapeutic effect, the longitudinal MRI image was used. Brain plasticity was assessed using morphometry. Patients underwent combined lower limb training with VR for four weeks (16–20 sessions).

Cortical thickness was compared with that of healthy controls. Clinically, improvements in balance, walking speed and muscle strength were achieved. All these results are based on neuroplasticity [16].

The problem that arises is the training time. The results of the study carried out on 21 patients (2017), which followed the effects of extending the robot-assisted treatment from 25 min to 50 min, support the superiority of motor function recovery [56]. Patients with an incomplete lesion (i.e., incomplete SCI for impairments in scales C and D) have a higher probability of regaining the ability to walk than those with partial sensory. The total rehabilitation rate for these patients is estimated to be approximately 75%. Patients who have less severe injuries, including lumbar and low thoracic injuries, can move with the assistance of braces and other supports. Furthermore, there are several variables that could affect these individuals’ prospects of mobility rehabilitation [57]. For tetraplegic sufferers, the most relevant factors are age, upper limb strength, motor rehabilitation period, and lower limb strength [58]. Age proved to be a strong predictor for walking rehabilitation in American Spinal Injury Association (ASIA) Impairment Scale C (AIS C) patients. In total, 80–90% of AIS C patients under the age of 50 will be capable of walking without assistance. However, this probability gradually decreases to 30–40% in patients over 50 [59]. Regeneration of lower limb functionality in patients with SCI is a primary concern in order to improve the autonomy and life quality of these patients [60]. About 66% of patients with SCI are paraplegic, and a significant percentage can restore specific mobility performance, particularly those with partial and low injuries [61]. 

The meta-analysis published in 2019 (114 studies), which aimed to synthesize the evidence of neurological recovery in 19,913 patients with posttraumatic SCI, presented the differences in recovery (depending on the scales used, ASIA and Frankel). It was found that ASIA C patients show a superior recovery, followed by ASIA B, D and A patients. Moreover, based on the severity of the lesion, patients with lumbar injuries recovered more quickly than those with cervical injury. The importance of the aetiology of the marrow injury (blunt or penetrating body) is also mentioned. However, the relationship with the type of treatment followed by the patients is not specified [62]. 

The advantage of using medical robots is the establishment of dynamic training programs. One of the robotic gait rehabilitation devices is Lokomat, a computer-controlled and electrically operated orthosis, to create a physiological gait pattern and restore lost proprioception [63]. Concerning the outcomes of robotic technology motor training in patients with acute incomplete SCI, Wirz et al. conducted a study that was published in 2011 that focused on whether SCI patients who experienced strong sensory-motor deficiencies following acute traumatic SCI (ASIA B and C) could benefit more from sustained Lokomat instruction than patients who had undergone the standard training methodology. The assumption was that patients with a serious but incomplete SCI who completed extensive Lokomat instruction would improve more quickly than those who finished the program as recommended by experts [56]. 

A further example would be balance-controlled robotic mobility systems like the REX (REX Bionics Ltd., Auckland 0627, New Zeeland), which can be utilized by patients with high SCI (up to C4/5 level) and completely replace their locomotor capacity. Consequently, these devices may be perfectly adapted for solely assistive applications. Additionally, portable exoskeletons designed to support leg mobility, like the ReWalk (Bionics Research Inc., Osaka, Japan) and the H2 (Technaid S.L., Osaka, Japan), are only suitable for use by patients who can achieve stability. These devices are significant because they can be managed using aid concepts, which may be more beneficial than other strategies for cognitive compensation and rehabilitation in stroke and paraplegic patients [63]. 

The study published in 2020 [64] on 13 patients who underwent the 6-min, and 30-min walking test supports the reduction of energy consumption by using the Robot (ReWalk) compared to knee-ankle-foot orthoses (KAFO). The monitored parameters for the assessment of energy consumption were heart rate, oxygen consumption, and metabolic equivalents. The study concluded that walking improved, as did walking distance with energy saving. The meta-analysis (14 studies) published in 2016 (*n* = 111 patients) evaluated the clinical results and the safety profile of using robotic technology therapy in SCI patients. ReWalk^TM^, Ekso^TM^, and Indego^®^ exoskeletons were implemented. The training program (walking, through obstacles, going up and down the stairs) followed by the patients consisted of 3 sessions/week, lasting 60–120 min. The number of weeks varied between 1 and 24 weeks. According to the meta-analysis findings, 76% of the patients were able to walk unassisted. Spasticity improved in 38% of patients. Perceived exertion was level 10 on the Borg scale [65]. The results obtained through training with Lokomat vs. autonomous exoskeletons were published in 2022 [66]. The meta-analysis included 12 studies from 2013–2021 and evaluated the patients’ walking performance. The conclusion of this meta-analysis was the ranking of autonomous devices in the first place and Lokomat in the second place regarding the effects on the performance of locomotion skills.

### 7.2. Effects on Elbow and RC Mobility

They aim to mobilize the elbow and the radiocarpal joint so that we get a functional hand. The degrees allowed by these devices are between 0 and 150 degrees for elbow flexion extension (to fulfil ADLs, freedom of movement between 30 and 120 degrees is necessary) and pronation-supination between −60 and 60 degrees [17]. For the recovery of the upper limbs, the Armeo Spring device is commonly recommended to patients with incomplete SCIs. An assessment by Zariffa et al. published in 2012 shows that the physiotherapist’s involvement is 25% during the treatment session. The study on 12 patients demonstrated that robot-assisted training has superior results in patients with incomplete injury and preserved function [67]. Most quadriplegic patients experience limitations in upper limb function that make them dependent on another person for most activities of daily living (ADL), which decreases the life quality for patients with quadriplegic SCI. A study (2019) conducted on 30 quadriplegic patients compared the results of traditional occupational rehabilitation with robotic technology intervention. (ASIA score from A to D). The treatment period lasted 5 weeks (3 sessions every 7 days, lasting 40 min). The robotic devices were Armeo Power (Hocoma AG, Volketswil, Switzerland), and AMADEO (Electron Ltd., Bristol, UK). The conclusion was that the therapeutic results on strength, sensitivity and prehension, the motor score (for the upper limbs), and the degree of independence is comparable [68].

Kadivar et al. published a pilot study (2011) in which they evaluated the feasibility of the device called Rice Wrist-S in the re-education of the upper limb in a young male with an incomplete cervical SCI. The use of this device in regaining the upper limb is supported by the case presentation [69]. 

In the rehabilitation of the upper limbs, the focus was on the proximal joints, whereby instituting an intensive and long-term treatment, with the help of robotic therapy, good results were obtained. The mobilization of the distal joints, so necessary for grasping movements, was less of a target of robotic treatment. MAHI Exo-II focuses on the distal joints. It is a high-performance rehabilitation exoskeleton that allows the implementation of complex control [70]. The Hand of Hope (HoH) (Rehab-Robotics Company Ltd., Hong Kong, China) is an exoskeleton for neurological recovery that enables people to re-establish hand motion; it consists of a system that utilizes serial link manipulators to actuate fingers. Among the earliest commercially accessible robotic arm rehabilitation devices was the HoH. Nevertheless, due to its restricted motion range and passive movement of the proximal interphalangeal (PIP), and the metacarpophalangeal (MCP) joints, the design should be improved, based on research by Ruddet al. published in 2019. The Festo Exo-Hand improved further in various areas, according to the same investigations, attributable to the application of a laser sintering procedure and 3D scanners. Both the system’s ability to regulate more degrees of freedom and its range of movement improved [71]. A static fixed, centered, end-effector limb recovery robot with numerous repeatable motions in three dimensions is called ReoGoTM (Motorika Medical, Caesarea, Israel). The ReoGo includes a real-time visual output display to provide exercises and games for the patient to complete. It also enables actions at the wrist, elbow (flexion/extension), and shoulder (i.e., flexion/extension, abduction/adduction, internal/external rotation). Furthermore, ReoGo was primarily utilized for stroke recovery of the upper limbs [72]. These upper extremity robotic devices are still being studied in patients with SCIs, and the technology improves yearly. With additional investigation and innovation, it may be possible to design interventions that sustain considerable functional increases by using more studies and validated performance indicators [73].

Currently, exoskeletons are being designed for anthropomorphic, lightweight, customized upper limbs that can be manipulated remotely [71].

## 8. Discussion

There are numerous benefits to using robotic techniques to help neurological patients recover. In addition to unloading the weight, facilitating the balance in orthostatism, and establishing a quasi-normal gait, long repetition of the exercise will affect the nervous system (taking into account the neuroplasticity). Proprioception is compromised in patients with SCI. For this reason, verticalization and initiation of walking, obtained with robotic devices, are essential for [63]. Moreover, with the Psychosocial Impact of the Assistive Device Scale Questionnaire, the implications of the robotics recovery process on the bio-psycho-social spectrum can be evaluated. It has been demonstrated that the use of exoskeletons has significant benefits in the recovery of motor function in disabled patients [51]. Many patients with SCI may preserve or regain the ability to walk, but endurance and walking speed may be impaired [74]. The main objective in recovering SCI is toning the muscles, either the upper or the lower train, to ensure as much independence as possible. Most of the training methods evaluated for increasing cardiac fitness are physical exercise or Computer-assisted Functional Electrical Stimulation (FES) training. It is proven that these methods increase tolerance and cardiac performance and improve the lipid and carbohydrate profile. The interaction between enhanced physical exercise and the condition of SCI has not yet been thoroughly assessed. [27].

The effects on walking parameters, resistance to effort, and spasticity were studied. The meta-analysis published in 2017 [75] includes the evaluation of 443 subjects, following the recovery effect of patients who benefited from robot-assisted gait training. The results showed an increase in walking independence and resistance to effort. Another meta-analysis published in 2020 (225 evaluated studies) that included 301 subjects supports the effectiveness of robotic technology in lowering stiffness, and improving muscle tone or walking, without affecting neuropathic pain [2]. The review of 16 studies, published in 2021, supports the benefits of training with Lokomat on walking (speed, distance, resistance) and mobility in patients with incomplete injuries and considers the need to continue studying the benefits of balance and complications associated with SCI [76]. 

There are few studies on cardiorespiratory response and metabolic consumption after training with robotic devices in patients with SCI. Data are published that show the reduced impact on the cardio-respiratory capacity given by the training performed passively by the exoskeleton on the treadmill compared to the use of autonomous exoskeletons, with the active participation of the subjects [30]. Furthermore, this study supports minimal increases in the energy cost obtained with the intensification of the effort. Therefore, a moderate effort of 150 min/week or 75 min of intense effort/week is recommended for patients with SCI. Chronic neurological disorders cause dysfunctions that affect daily activities and socio-professional life [77]. 

The patient’s gait and upper limb function recovery is a primary goal [78]. Unfortunately, many affected individuals fail to recover upper and lower extremity function despite prolonged rehabilitative treatment. Because of this, in recent years, clinicians, physiotherapists and engineers have collaborated to help patients recover better. The role of robotic devices is to reduce the effort of the physiotherapist [79]. The recovery of the motor function of the upper limbs is essential for obtaining the independence of patients with tetraplegia after SCI; an attempt was made to design some devices that would support the arm and stimulate the mobilization of the upper limb [80]. Due to the complexity of the upper limb, the technology used in its recovery is increasingly complex to allow the mobilization of certain degrees of movement [81]. 

Robotic walking orthoses and exoskeletal systems have been developed to obtain the highest degree of independence, enabling patient mobilization off the treadmill [68]. Exoskeletons for the lower extremities feature motors that control motions over these joints to support flexion and extension. Patients with SCI can benefit from Rex^®^, EKSO™ and ReWalk^®^ to improve their mobility (walking, climbing stairs) [82,83]. The Lokomat rehabilitation robot enables automatic treadmill training for patients with lower limb mobility impairments [84]. Patients with severe SCI impairment can conduct efficient gait exercise without any particular health consequences due to robotic-assisted gait training (RAGT) [85]. The results of training with RAGT (Table 3), the lesion level and the complete/incomplete type of the lesion.

The potential of tDCS to possibly improve the clinical benefit of robot-assisted rehabilitation in chronic SCI patients has been assessed in a recent finding. Participants in the experiment performed robot-assisted mobility therapy after 20 min of either active or sham tDCS. The investigation was randomized, double-blind, and sham-controlled. Participants with incomplete cervical lesions showed more significant improvements in the arm and hand functional scores on the Jebsen-Taylor Hand Function Test when compared to patients who received sham tDCS, according to Yozbatiran et al. [88]. Another complication of SCI that may benefit from RT is osteoporosis. The lack of use of the limbs seems to be an essential factor in the development, but the pathophysiology is not fully known. The management of SCI patients should include early bone medical assessment and continuous observation.

In addition to using vitamin supplements and bisphosphonates, robot-assisted exercise has been used to prevent osteoporosis but did not show an improvement in bone mineral density [89]. RAGT training makes the patient’s energy consumption more efficient by unloading the weight and initiating motor activity, but it additionally requires the cardio-respiratory system compared to free walking. This fact involves the evaluation of the patient before the initiation of the training. Furthermore, the intensity of the effort and the duration must be established according to the cardio-respiratory reserve [90]. 

### 8.1. Working Group on Artificial Intelligence and Robotics

The European Parliament’s Legal Affairs Committee established the Working Group on regulatory issues concerning robotics innovation to reflect on legal aspects and especially to pave the way for drafting civil law rules in connection with robotics and artificial intelligence. Its mission is to stimulate the reflection of Members on these issues by facilitating specific information, providing an exchange of views with experts from many fields of academic expertise, and enabling Members to conduct an in-depth analysis/examination of the challenges and prospects at stake. The input gathered by the Working Group will be put forward as a basis for future legislative activities.

### 8.2. Quality of Life of Patients with SCI

If the damage to the marrow is below the T12 level, we can speak of a functional gait. By verticalizing the patient, spasticity is reduced, the risk of thrombosis, pressure ulcers and pressure ulcers, bone mass reduction, and the possibilities of bladder and bowel recovery increase [91]. Social reintegration is an essential and determining process for increasing the quality of life of people with disabilities. It is necessary to be active and to carry out activities corresponding to age, educational level, and functional deficit. A study (2021) conducted on 62 patients with SCI with a lesion level at Th6-Th12 showed that only 50% were active and gave particular importance to their interpersonal relationships [92]. 

Most studies evaluate the recovery of motor function, mainly walking, walking speed, and less the effects on the other systems.

## 9. Conclusions and Future Directions

RAGT allows SCI patients to perform effective, prolonged locomotor training without specific side effects. The benefits consist, first of all, of increasing mobility, improving walking, and reducing spasticity.

Positive effects are also obtained on the complications associated with SCI at the cardiorespiratory, digestive, bone, and mental levels, causing an increase in the quality of life. Robotic therapy, with complex recovery treatment and specific medication, increases the chances of neuro-motor recovery. In addition, the exoskeletons allow patients more autonomy and better social integration.

A proper cardio-respiratory assessment of the patient with SCI, establishing the time of use of the walking device, and the intensity of the effort bring significant benefits to the neurological recovery. In addition, using mobile exoskeletons will determine greater independence of patients, with effects on their psyche.

## Figures and Tables

**Table 1:** Functional outcomes following complete SCIs.

Neurological Level of Injury	Functional Deficit of the Patient	Remaining Functional
C3	The patient cannot breathe spontaneously and needs ventilator support.	-
C4	The global functional deficit requires an electric chair controlled by breathing, tongue or jaw, and static wrist orthosis for forearm and hand posture.	Impairment of diaphragm function, necessary endotracheal intubation, and mechanical ventilation
C5	Totally dependent on transfers and ADL	Breathe spontaneously Does elbow flexion, Joystick-controlled wheelchair
C6	Assisted transfer, Needing an electric chair for longer distances Requirements of intermittent probing of the urinary bladder	Active wrist extension Independent in activities such as nutrition, grooming, hygiene, and dressing of the upper train
C6-C7	Help for dressing the lower train Needing a manual wheelchair	Elbow extension (C7), finger flexion (C8) Quasi-independent for transfers
T11-T12	Needing manual chair, Neurogenic bowel Neurogenic bladder	Independence to perform ADLs Walking with orthotics is initiated
L1-L2	Traveling with a chair over long distances	Totally independent, capable to move short distances with walking orthotics, Can do knee flexion and partial plantar dorsiflexion Presenting bladder and intestinal sphincter control
Sub L5	-	Total independence

C, cervical; T, thoracal; L, lumbar; ADL, activity of daily living.

**Table 2:** Dependency degrees in correlation with the severity of the injury.

Neurological Level of Injury	Transfers	Manual Wheelchair(W/C) Skills	Ambulation
C1-C4	100% assistance from another person	W/C propelled by another person	No functional ambulation
C5			
C6	80–100% assistance from another person	W/C propelled by another person outdoors, some are independent indoors	
C7-C8	Independent in some cases	Independent indoors, sometimes outdoors, some need assistance with unlevel terrain	
T1-T9	Independent	Independent indoors and outdoors on the level and unloved terrain	Functional ambulation is not typical
T10-L1			Patients may be able to walk using KAFOs
L2-S5			Patients may be able to walk using KAFOs, or AFOs and forearm crutches or cane(s)

C, cervical; T, thoracal; L, lumbar; KAFOs, Knee-Ankle-Foot Orthosis; AFOs, Ankle-Foot Orthosis.

**Table 3:** Future ambulatory prognosis studies results.

Characteristics	Patients, Diagnostics	Outcomes
Complete vs. incomplete and paraplegia vs. tetraplegia	246 patients with complete paraplegia, incomplete paraplegia and tetraplegia [63]	Complete paraplegia: 5% ambulators at one year Incomplete paraplegia: 76% ambulators at one year Incomplete tetraplegia: 46%ambulators at one year
ASIA classification	80 with incomplete paraplegia, ASIA C and D [57]	About 75% of persons achieve walking ability
Initial ASIA classification and age	105 patients with incomplete tetraplegia ASIA C and D [86]	Tetraplegia, ASIA C, <50 years old 91% ambulators by discharge from rehabilitation ward Tetraplegia, ASIA C, >50 years, 42% ambulators by discharge Tetraplegia, ASIA D, 100% ambulators by discharge, regardless of age.
Pinprick sensations spared in lower extremities	97 patients with ASIA B paraplegia and tetraplegia as a result of trauma [59]	Pinprick sensation spared in >50% of L2–S1 dermatomes: 40% able to walk independently >150 feet 1 year after injury.
Preservation of iliopsoas muscle strength at 30 days after injury	54 patients with incomplete paraplegia due to trauma [87]	All with >2/5 initial hip flexor or knee extensor strength were ambulators at one year.

ASIA, American Spinal Injury Association.

## Data Availability

Not applicable.

## References

[1] Nas K., Yazmalar L., Şah V., Aydın A., Öneş K. (2015). Rehabilitation of spinal cord injuries. World J. Orthop..

[2] Fang C.Y., Tsai J.L., Li G.S., Lien A.S., Chang Y.J. (2020). Effects of Robot-Assisted Gait Training in Individuals with Spinal Cord Injury: A Meta-analysis. Biomed. Res. Int..

[3] Tamburella F., Lorusso M., Tramontano M., Fadlun S., Masciullo M., Scivoletto G. (2022). Overground robotic training effects on walking and secondary health conditions in individuals with spinal cord injury: Systematic review. J. Neuroeng. Rehabil..

[4] Jain N.B., Ayers G.D., Peterson E.N., Harris M.B., Morse L., O’Connor K.C., Garshick E. (2015). Traumatic spinal cord injury in the United States, 1993–2012. JAMA.

[5] Holanda L.J., Silva P.M.M., Amorim T.C., Lacerda M.O., Simão C.R., Morya E. (2017). Robotic assisted gait as a tool for rehabilitation of individuals with spinal cord injury: A systematic review. J. Neuroeng. Rehabil..

[6] Alizadeh A., Dyck S.M., Karimi-Abdolrezaee S. (2019). Traumatic Spinal Cord Injury: An Overview of Pathophysiology, Models and Acute Injury Mechanisms. Front. Neurol..

[7] Page M.J., McKenzie J.E., Bossuyt P.M., Boutron I., Hoffmann T.C., Mulrow C.D., Shamseer L., Tetzlaff J.M., Akl E.A., Brennan S.E. (2021). The PRISMA 2020 statement: An updated guideline for reporting systematic reviews. Rev. Esp. Cardiol..

[8] Devivo M.J. (2012). Epidemiology of traumatic spinal cord injury: Trends and future implications. Spinal Cord.

[9] Jacobs P.L., Nash M.S. (2004). Exercise recommendations for individuals with spinal cord injury. Sports Med..

[10] Uivarosan D., Tit D.M., Iovan C., Nistor-Cseppento D.C., Endres L., Lazar L., Sava C., Sabau A.M., Buhas C., Moleriu L.C. (2019). Effects of combining modern recovery techniques with neurotrophic medication and standard treatment in stroke patients. Sci. Total Environ..

[11] Uivarosan D., Abdel-Daim M.M., Endres L., Purza L., Iovan C., Bungau S., Furau C.G., Tit D.M. (2018). Effects of a proteic swine extract associated to recovery treatment on functional independence and quality of life in patients post stroke. Farmacia.

[12] Esquenazi A., Talaty M., Jayaraman A. (2017). Powered Exoskeletons for Walking Assistance in Persons with Central Nervous System Injuries: A Narrative Review. PM&R.

[13] Birch N., Graham J., Priestley T., Heywood C., Sakel M., Gall A., Nunn A., Signal N. (2017). Results of the first interim analysis of the RAPPER II trial in patients with spinal cord injury: Ambulation and functional exercise programs in the REX powered walking aid. J. Neuroeng. Rehabil..

[14] Yıldırım M.A., Öneş K., Gökşenoğlu G. (2019). Early term effects of robotic assisted gait training on ambulation and functional capacity in patients with spinal cord injury. Turk. J. Med. Sci..

[15] Mekki M., Delgado A.D., Fry A., Putrino D., Huang V. (2018). Robotic Rehabilitation and Spinal Cord Injury: A Narrative Review. Neurotherapeutics.

[16] Villiger M., Grabher P., Hepp-Reymond M.C., Kiper D., Curt A., Bolliger M., Hotz-Boendermaker S., Kollias S., Eng K., Freund P. (2015). Relationship between structural brainstem and brain plasticity and lower-limb training in spinal cord injury: A longitudinal pilot study. Front. Hum. Neurosci..

[17] Copaci D., Arias J., Moreno L., Blanco D., Olaru A.D. (2022). Shape Memory Alloy (SMA)-Based Exoskeletons for Upper Limb Rehabilitation. Rehabilitation of the Human Bone-Muscle System.

[18] Rodríguez-Fernández A., Lobo-Prat J., Font-Llagunes J.M. (2021). Systematic review on wearable lower-limb exoskeletons for gait training in neuromuscular impairments. J. Neuroeng. Rehabil..

[19] Federici S., Meloni F., Bracalenti M., De Filippis M.L. (2015). The effectiveness of powered, active lower limb exoskeletons in neurorehabilitation: A systematic review. NeuroRehabilitation.

[20] De Araújo A.V.L., Neiva J.F.O., Monteiro C.B.M., Magalhães F.H. (2019). Efficacy of Virtual Reality Rehabilitation after Spinal Cord Injury: A Systematic Review. Biomed. Res. Int..

[21] Prasad S., Aikat R., Labani S., Khanna N. (2018). Efficacy of Virtual Reality in Upper Limb Rehabilitation in Patients with Spinal Cord Injury: A Pilot Randomized Controlled Trial. Asian Spine J..

[22] Sengupta M., Gupta A., Khanna M., Rashmi Krishnan U.K., Chakrabarti D. (2020). Role of Virtual Reality in Balance Training in Patients with Spinal Cord Injury: A Prospective Comparative Pre-Post Study. Asian Spine J..

[23] Polanía R., Nitsche M.A., Ruff C.C. (2018). Studying and modifying brain function with non-invasive brain stimulation. Nat. Neurosci..

[24] Fregni F., Pascual-Leone A. (2007). Technology Insight: Noninvasive brain stimulation in neurology—Perspectives on the therapeutic potential of rTMS and tDCS. Nat. Clin. Pract. Cardiovasc. Med..

[25] Krishnan C., Santos L., Peterson M.D., Ehinger M. (2015). Safety of noninvasive brain stimulation in children and adolescents. Brain Stimul..

[26] Yang R., Guo L., Wang P., Huang L., Tang Y., Wang W., Chen K., Ye J., Lu C., Wu Y. (2014). Epidemiology of spinal cord injuries and risk factors for complete injuries in Guangdong, China: A retrospective study. PLoS ONE.

[27] Warburton D.E., Eng J.J., Krassioukov A., Sproule S. (2007). Cardiovascular Health and Exercise Rehabilitation in Spinal Cord Injury. Top. Spinal Cord Inj. Rehabil..

[28] Harman K.A., DeVeau K.M., Squair J.W., West C.R., Krassioukov A.V., Magnuson D.S.K. (2021). Effects of early exercise training on the severity of autonomic dysreflexia following incomplete spinal cord injury in rodents. Physiol. Rep..

[29] Rodriguez B., Santiago-Tovar P., Guerrero-Godinez M., Garcia Vences E.E. (2020). Rehabilitation Therapies in Spinal Cord Injury Patients. Paraplegia.

[30] Evans N., Hartigan C., Kandilakis C., Pharo E., Clesson I. (2015). Acute Cardiorespiratory and Metabolic Responses During Exoskeleton-Assisted Walking Overground Among Persons with Chronic Spinal Cord Injury. Top. Spinal Cord Inj. Rehabil..

[31] Ravensbergen H.J., de Groot S., Post M.W., Slootman H.J., van der Woude L.H., Claydon V.E. (2014). Cardiovascular function after spinal cord injury: Prevalence and progression of dysfunction during inpatient rehabilitation and 5 years following discharge. Neurorehabil. Neural. Repair..

[32] Lee E.S., Joo M.C. (2017). Prevalence of Autonomic Dysreflexia in Patients with Spinal Cord Injury above T6. Biomed. Res. Int..

[33] Turiel M., Sitia S., Cicala S., Magagnin V., Bo I., Porta A., Caiani E., Ricci C., Licari V., De Gennaro Colonna V. (2011). Robotic treadmill training improves cardiovascular function in spinal cord injury patients. Int. J. Cardiol..

[34] Escalona M.J., Brosseau R., Vermette M., Comtois A.S., Duclos C., Aubertin-Leheudre M., Gagnon D.H. (2018). Cardiorespiratory demand and rate of perceived exertion during overground walking with a robotic exoskeleton in long-term manual wheelchair users with chronic spinal cord injury: A cross-sectional study. Ann. Phys. Rehabil. Med..

[35] Elliott S., Krassioukov A. (2006). Malignant autonomic dysreflexia in spinal cord injured men. Spinal Cord.

[36] Taweel W.A., Seyam R. (2015). Neurogenic bladder in spinal cord injury patients. Res. Rep. Urol..

[37] Gifre L., Vidal J., Carrasco J.L., Muxi A., Portell E., Monegal A., Guañabens N., Peris P. (2016). Denosumab increases sublesional bone mass in osteoporotic individuals with recent spinal cord injury. Osteoporos. Int..

[38] Tit D.M., Bungau S., Iovan C., Nistor Cseppento D.C., Endres L., Sava C., Sabau A.M., Furau G., Furau C. (2018). Effects of the hormone replacement therapy and of soy isoflavones on bone resorption in postmenopause. J. Clin. Med..

[39] Ţiţ D.M., Pallag A., Iovan C., Furău G., Furău C., Bungău S. (2017). Somatic-vegetative Symptoms Evolution in Postmenopausal Women Treated with Phytoestrogens and Hormone Replacement Therapy. Iran. J. Public Health.

[40] Abdelrahman S., Ireland A., Winter E.M., Purcell M., Coupaud S. (2021). Osteoporosis after spinal cord injury: Aetiology, effects and therapeutic approaches. J. Musculoskelet. Neuronal Interact..

[41] Frotzler A., Berger M., Knecht H., Eser P. (2008). Bone steady-state is established at reduced bone strength after spinal cord injury: A longitudinal study using peripheral quantitative computed tomography (pQCT). Bone.

[42] Bauman W.A., Spungen A.M., Wang J., Pierson R.N., Schwartz E. (1999). Continuous loss of bone during chronic immobilization: A monozygotic twin study. Osteoporos. Int..

[43] Szollar S.M., Martin E.M., Parthemore J.G., Sartoris D.J., Deftos L.J. (1997). Densitometric patterns of spinal cord injury associated bone loss. Spinal Cord.

[44] Demirel G., Yilmaz H., Paker N., Onel S. (1998). Osteoporosis after spinal cord injury. Spinal Cord.

[45] Morse L.R., Sudhakar S., Danilack V., Tun C., Lazzari A., Gagnon D.R., Garshick E., Battaglino R.A. (2012). Association between sclerostin and bone density in chronic spinal cord injury. J. Bone Miner. Res..

[46] Eser P., Frotzler A., Zehnder Y., Schiessl H., Denoth J. (2005). Assessment of anthropometric, systemic, and lifestyle factors influencing bone status in the legs of spinal cord injured individuals. Osteoporos. Int..

[47] Berlowitz D.J., Wadsworth B., Ross J. (2016). Respiratory problems and management in people with spinal cord injury. Breathe.

[48] Randelman M., Zholudeva L.V., Vinit S., Lane M.A. (2021). Respiratory Training and Plasticity After Cervical Spinal Cord Injury. Front. Cell. Neurosci..

[49] Zhou H.L. (2017). Treatment of erectile dysfunction in patients with spinal cord injury. Zhonghua Nan Ke Xue.

[50] Cadel L., DeLuca C., Hitzig S.L., Packer T.L., Lofters A.K., Patel T., Guilcher S.J.T. (2020). Self-management of pain and depression in adults with spinal cord injury: A scoping review. J. Spinal Cord Med..

[51] Fundarò C., Giardini A., Maestri R., Traversoni S., Bartolo M., Casale R. (2018). Motor and psychosocial impact of robot-assisted gait training in a real-world rehabilitation setting: A pilot study. PLoS ONE.

[52] Stokes S., Drozda M., Lee C. (2022). The past, present, and future of traumatic spinal cord injury therapies: A review. Bone Jt. Open.

[53] Pozeg P., Palluel E., Ronchi R., Solcà M., Al-Khodairy A.W., Jordan X., Kassouha A., Blanke O. (2017). Virtual reality improves embodiment and neuropathic pain caused by spinal cord injury. Neurology.

[54] Austin P.D., Siddall P.J. (2021). Virtual reality for the treatment of neuropathic pain in people with spinal cord injuries: A scoping review. J. Spinal Cord Med..

[55] Spiess M.R., Steenbrink F., Esquenazi A. (2018). Getting the Best Out of Advanced Rehabilitation Technology for the Lower Limbs: Minding Motor Learning Principles. PM&R.

[56] Wirz M., Mach O., Maier D., Benito-Penalva J., Taylor J., Esclarin A., Dietz V. (2017). Effectiveness of Automated Locomotor Training in Patients with Acute Incomplete Spinal Cord Injury: A Randomized, Controlled, Multicenter Trial. J. Neurotrauma.

[57] Alcobendas-Maestro M., Esclarín-Ruz A., Casado-López R.M., Muñoz-González A., Pérez-Mateos G., González-Valdizán E., Martín J.L. (2012). Lokomat robotic-assisted versus overground training within 3 to 6 months of incomplete spinal cord lesion: Randomized controlled trial. Neurorehabilit. Neural Repair.

[58] Subbarao J.V. (1996). Ambulation in spinal cord injured patients-options: Where do we stand?. J. Spinal Cord Med..

[59] Burns S.P., Golding D.G., Rolle W.A., Graziani V., Ditunno J.F. (1997). Recovery of ambulation in motor-incomplete tetraplegia. Arch. Phys. Med. Rehabil..

[60] Van Hedel H.J., Dietz V. (2010). Rehabilitation of locomotion after spinal cord injury. Restor. Neurol. Neurosci..

[61] Burns A.S., Marino R.J., Flanders A.E., Flett H. (2012). Clinical diagnosis and prognosis following spinal cord injury. Handb. Clin. Neurol..

[62] Khorasanizadeh M., Yousefifard M., Eskian M., Lu Y., Chalangari M., Harrop J.S., Jazayeri S.B., Seyedpour S., Khodaei B., Hosseini M. (2019). Neurological recovery following traumatic spinal cord injury: A systematic review and meta-analysis. J. Neurosurg. Spine.

[63] Domingo A., Lam T. (2014). Reliability and validity of using the Lokomat to assess lower limb joint position sense in people with incomplete spinal cord injury. J. Neuroeng. Rehabil..

[64] Kwon S.H., Lee B.S., Lee H.J., Kim E.J., Lee J.A., Yang S.P., Kim T.Y., Pak H.R., Kim H.K., Kim H.Y. (2020). Energy Efficiency and Patient Satisfaction of Gait With Knee-Ankle-Foot Orthosis and Robot (ReWalk)-Assisted Gait in Patients With Spinal Cord Injury. Ann. Rehabil. Med..

[65] Miller L.E., Zimmermann A.K., Herbert W.G. (2016). Clinical effectiveness and safety of powered exoskeleton-assisted walking in patients with spinal cord injury: Systematic review with meta-analysis. Med. Devices.

[66] Zhang L., Lin F., Sun L., Chen C. (2022). Comparison of Efficacy of Lokomat and Wearable Exoskeleton-Assisted Gait Training in People with Spinal Cord Injury: A Systematic Review and Network Meta-Analysis. Front. Neurol..

[67] Zariffa J., Kapadia N., Kramer J.L., Taylor P., Alizadeh-Meghrazi M., Zivanovic V., Willms R., Townson A., Curt A., Popovic M.R. (2012). Feasibility and efficacy of upper limb robotic rehabilitation in a subacute cervical spinal cord injury population. Spinal Cord.

[68] Jung J.H., Lee H.J., Cho D.Y., Lim J.E., Lee B.S., Kwon S.H., Kim H.Y., Lee S.J. (2019). Effects of Combined Upper Limb Robotic Therapy in Patients with Tetraplegic Spinal Cord Injury. Ann. Rehabil. Med..

[69] Kadivar Z., Sullivan J.L., Eng D.P., Pehlivan A.U., O’Malley M., Yozbatiran N., Francisco G.E. (2011). Robotic Training and Kinematic Analysis of Arm and Hand after Incomplete Spinal Cord Injury: A Case Study, Proceedings of the 2011 IEEE International Conference on Rehabilitation Robotics, Zurich, Switzerland, 29 June–1 July 2011.

[70] French J.A., Rose C.G., O’Malley M.K. (2014). System Characterization of MAHI EXO-II: A Robotic Exoskeleton for Upper Extremity Rehabilitation. Proc. ASME Dyn. Syst. Control. Conf..

[71] Rudd G., Daly L., Jovanovic V., Cuckov F. (2019). A Low-Cost Soft Robotic Hand Exoskeleton for Use in Therapy of Limited Hand–Motor. Appl. Sci..

[72] Takebayashi T., Takahashi K., Amano S., Uchiyama Y., Gosho M., Domen K., Hachisuka K. (2018). Assessment of the Efficacy of ReoGo-J Robotic Training Against Other Rehabilitation Therapies for Upper-Limb Hemiplegia After Stroke: Protocol for a Randomized Controlled Trial. Front. Neurol..

[73] Mulcahey M.J., Hutchinson D., Kozin S. (2007). Assessment of upper limb in tetraplegia: Considerations in evaluation and outcomes research. J. Rehabil. Res. Dev..

[74] Kim C.M., Eng J.J., Whittaker M.W. (2004). Level walking and ambulatory capacity in persons with incomplete spinal cord injury: Relationship with muscle strength. Spinal Cord.

[75] Cheung E.Y.Y., Ng T.K.W., Yu K.K.K., Kwan R.L.C., Cheing G.L.Y. (2017). Robot-Assisted Training for People With Spinal Cord Injury: A Meta-Analysis. Arch. Phys. Med. Rehabil..

[76] Alashram A.R., Annino G., Padua E. (2021). Robot-assisted gait training in individuals with spinal cord injury: A systematic review for the clinical effectiveness of Lokomat. J. Clin. Neurosci..

[77] Cseppento C.D.N., Iovanovici I., Andronie-Cioara F.L., Tarce A.G., Bochiș C.F., Bochiș S.A., Gabriela D.B.G.D.B. (2022). The recovery management of patients with operated extramedullary spinal arteriovenous fistula, evolution and socio-professional reintegration: Case report and review of the literature. Balneo PRM Res. J..

[78] Roberts T.T., Leonard G.R., Cepela D.J. (2017). Classifications In Brief: American Spinal Injury Association (ASIA) Impairment Scale. Clin. Orthop. Relat. Res..

[79] Sale P., Franceschini M., Waldner A., Hesse S. (2012). Use of the robot assisted gait therapy in rehabilitation of patients with stroke and spinal cord injury. Eur. J. Phys. Rehabil. Med..

[80] Schwartz I., Sajina A., Neeb M., Fisher I., Katz-Luerer M., Meiner Z. (2011). Locomotor training using a robotic device in patients with subacute spinal cord injury. Spinal Cord.

[81] Klamroth-Marganska V., Blanco J., Campen K., Curt A., Dietz V., Ettlin T., Felder M., Fellinghauer B., Guidali M., Kollmar A. (2014). Three-dimensional, task-specific robot therapy of the arm after stroke: A multicentre, parallel-group randomised trial. Lancet Neurol..

[82] Sale P., Russo E.F., Russo M., Masiero S., Piccione F., Calabrò R.S., Filoni S. (2016). Effects on mobility training and de-adaptations in subjects with Spinal Cord Injury due to a Wearable Robot: A preliminary report. BMC Neurol..

[83] Esquenazi A., Talaty M., Packel A., Saulino M. (2012). The ReWalk powered exoskeleton to restore ambulatory function to individuals with thoracic-level motor-complete spinal cord injury. Am. J. Phys. Med. Rehabil..

[84] Vilimovsky T., Chen P., Hoidekrova K., Slavicek O., Harsa P. (2022). Prism Adaptation Treatment Predicts Improved Rehabilitation Responses in Stroke Patients with Spatial Neglect. Healthcare.

[85] Zeilig G., Weingarden H., Zwecker M., Dudkiewicz I., Bloch A., Esquenazi A. (2012). Safety and tolerance of the ReWalk™ exoskeleton suit for ambulation by people with complete spinal cord injury: A pilot study. J. Spinal Cord Med..

[86] Waters R.L. (1996). Functional prognosis of spinal cord injuries. J. Spinal Cord Med..

[87] Oleson C.V., Burns A.S., Ditunno J.F., Geisler F.H., Coleman W.P. (2005). Prognostic value of pinprick preservation in motor complete, sensory incomplete spinal cord injury. Arch. Phys. Med. Rehabil..

[88] Yozbatiran N., Keser Z., Davis M., Stampas A., O’Malley M.K., Cooper-Hay C., Frontera J., Fregni F., Francisco G.E. (2016). Transcranial direct current stimulation (tDCS) of the primary motor cortex and robot-assisted arm training in chronic incomplete cervical spinal cord injury: A proof of concept sham-randomized clinical study. NeuroRehabilitation.

[89] Gordon K.E., Wald M.J., Schnitzer T.J. (2013). Effect of parathyroid hormone combined with gait training on bone density and bone architecture in people with chronic spinal cord injury. PM&R.

[90] Lefeber N., Swinnen E., Kerckhofs E. (2017). The immediate effects of robot-assistance on energy consumption and cardiorespiratory load during walking compared to walking without robot-assistance: A systematic review. Disabil. Rehabil. Assist. Technol..

[91] Guest R.S., Klose K.J., Needham-Shropshire B.M., Jacobs P.L. (1997). Evaluation of a training program for persons with SCI paraplegia using the Parastep 1 ambulation system: Part 4. Effect on physical self-concept and depression. Arch. Phys. Med. Rehabil..

[92] Filipcic T., Sember V., Pajek M., Jerman J. (2021). Quality of Life and Physical Activity of Persons with Spinal Cord Injury. Int. J. Environ. Res. Public Health.

